# Personal

**Published:** 1916-02

**Authors:** 


### PERSONAL.

Announcement of removal, travel, and other matters of interest are requested. Please report errors in the listing of any physician in the State and other directories, that we may co-operate with the State Society in securing a correct list.

Our associate editor, Dr. Grover W. Wende of Buffalo, has presented to the Buffalo Historical Society, an oil portrait of his brother, the late Ernest Wende, long Health Commissioner of Buffalo. The artist is L. Bernice.

Dr. Horace S. Hutchins of Batavia observed his 87th birthday Jan. 5. He is still in practice.

Dr. Pemberton Lundy of N. Tonawanda has been appointed to the local board of health.

Dr. David F. Wheeler, formerly of Buffalo, has been decorated with the War Cross by the French government, for valorous conduct in the Foreign Legion, on Sept. 28, when he was severely wounded.

Dr. A. H. Bradbury of Grand Island was severely injured by the skidding of his automobile, while responding to a sick call, Dec. 27.

---

Dr. Lucien Howe of Buffalo, addressed the Hamburg Social Center on Preparedness, Jan. 3.

---

Dr. Max C. Breuer of Buffalo returned from Lake Placid, Jan. 4.

---

Dr. S. S. Green of Buffalo, celebrated his Golden Wedding and 77th birthday, Jan. 6.

---

Dr. Harvey L. Brown has assumed charge of the Naval Recruiting Office at Buffalo, vice Dr. J. J. A. McMullen who has left for the Philippines.

---

Dr. Eugene E. Webster of Woodhull, has been elected chairman of the Steuben Co. Board of Supervisors.

---

Dr. H. J. Wynkoop of Bath has been appointed Physician to the Steuben Co. Jail.

---

Dr. Douglass Smith of Bath has been appointed Physician to the Steuben Co. Poor Farm.

---

Dr. George E. Ellis, Health Officer of Dunkirk, has had his salary raised from $70 to $80 a month.

---

Dr. Harry B. Lyon of Dunkirk has been appointed Secretary of the Board of Health.

---

Dr. John Parmenter of High Acres Farm, Geneva, visited in Buffalo late in January.

---

Dr. Charles F. Howard of Buffalo sustained a fracture of the left scapula, in an automobile collision, January 23.

---

Dr. Charles J. Mengis of Buffalo, has moved to 23 W. Oakwood Place.

---

Dr. Grace Ilahi-Baksh, M. D., University of Buffalo 1913, has been engaged by the Lutheran Church of the Redeemer as a medical missionary. She is now an interne in the German Deaconess Hospital, and will leave about August 1 to serve in the Lutheran Hospital at Rajahmundry, in the Madras Presidency of India. Her parents are at present engaged in missionary service in their native country, India, her mother being also a physician.

